# Protocol for a systematic review of the use of narrative storytelling and visual-arts-based approaches as knowledge translation tools in healthcare

**DOI:** 10.1186/2046-4053-2-19

**Published:** 2013-03-20

**Authors:** Shannon D Scott, Pamela Brett-MacLean, Mandy Archibald, Lisa Hartling

**Affiliations:** 1 Faculty of Nursing, University of Alberta, Level 3, Edmonton Clinic Health Academy, Edmonton, Alberta, T6G 1C9, Canada; 2 Arts & Humanities in Health & Medicine Program, Faculty of Medicine & Dentistry, University of Alberta, 1-128 Katz Group Centre for Pharmacy and Health Research, Edmonton, Alberta, T6G 2X8, Canada; 3 Alberta Research Centre for Health Evidence, Department of Pediatrics, Faculty of Medicine and Dentistry, University of Alberta, Level 4, Edmonton Clinic Health Academy, Edmonton, Alberta, T6G 1C9, Canada

**Keywords:** Knowledge translation, Narrative, Research use, Storytelling, Systematic review, Visual art

## Abstract

**Background:**

The arts are powerful, accessible forms of communication that have the potential to impart knowledge by attracting interest and developing meaningful connections. Knowledge translation aims to reduce the ‘evidence-practice’ gap by developing, implementing and evaluating strategies designed to enhance awareness and promote behavior change congruent with research evidence. Increasingly, innovative approaches such as narrative storytelling and other arts-based interventions are being investigated to bridge the growing gap between practice and research. This study is the first to systematically identify and synthesize current research on narrative storytelling and visual art to translate and disseminate health research.

**Methods:**

A health research librarian will develop and implement search strategies designed to identify relevant evidence. Studies will be included if they are primary research employing narrative storytelling and/or visual art as a knowledge translation strategy in healthcare. Two reviewers will independently perform study selection, quality assessment, and data extraction using standard forms. Disagreements will be resolved through discussion or third party adjudication. Data will be grouped and analyzed by research design, type of knowledge translation strategy (that is, a narrative or visual-arts-based approach), and target audience. An overall synthesis across all studies will be conducted.

**Discussion:**

The findings from this research project will describe the ‘state of the science’ regarding the use of narrative storytelling and visual art as knowledge translation strategies. This systematic review will provide critical information for: (1) researchers conducting knowledge translation intervention studies; (2) nursing, medicine, and allied healthcare professionals; (3) healthcare consumers, including patients and families; and (4) decision makers and knowledge users who are charged to increase use of the latest research in healthcare settings.

## Introduction

Storytelling [1] and visual art are powerful, ancient means of embodying and reinforcing ‘socially shared significances’ [2], that can cut across age, culture, language, literacy, and gender barriers. Independently and together, storytelling and visual art have the potential to create shared, embodied understandings through imparting knowledge. One mechanism through which they may work is by attracting and sustaining interest, and engaging recipients in making meaningful connections [3]. More importantly storytelling and visual art are highly accessible modalities that do not require specialized knowledge and skills to connect with or derive meaning from. Although recognized as an effective and engaging means of communication [4,5], storytelling and visual art have not been utilized to their full potential in the healthcare environment [6]; however, there is increasing interest in their power to motivate, communicate, heal [7-11], and engage with multiple audiences [12-15].

## Background

Knowledge translation (KT) is a dynamic and iterative process [16]. It seeks to mobilize best-practice evidence to guide decisions in healthcare and is an integral component of the evidence-based practice movement. Despite current emphasis on evidence-based practice, there remains a growing gap between ‘what is known’ and ‘what is done’ [17,18]. KT aims to reduce the ‘evidence-practice’ gap by developing, implementing, and evaluating strategies designed to enhance awareness and promote behavior change congruent with research evidence [19]. This behavior change can take place in many audiences including healthcare professionals, decision makers and healthcare consumers [20]. An emerging area of KT science involves developing and evaluating effective interventions for healthcare consumers (for example, patient-mediated interventions), however the bulk of effort in the field has been dedicated to strategies that have been deemed effective in translating knowledge to healthcare professionals (for example, decision-making guides, reminders, educational materials). Concomitantly, there is a move in healthcare towards an integrative, collaborative, and patient-centered model of care that requires strategies be developed to: (1) promote knowledge uptake and dissemination, (2) be suitable for multiple audiences (for example, healthcare professionals, healthcare consumers, decision makers), and (3) account for patient preferences in care provision and patient involvement in decision-making. For instance, interventions such as narratives designed to elicit the consumers’ experience of, and preferences pertaining to, health-illness experiences, are increasingly seen as central to patient-centered care provision and collaboration [21].

A growing awareness of the complex nature of decision-making in healthcare for both provider and recipient and the related health-illness experience has prompted a movement toward exploring innovative approaches to transfer research and communicate with multiple audiences [12]. Specifically, the use of narrative storytelling and other arts-based approaches (such as visual art) as communication and teaching tools are increasingly suggested and utilized as innovative approaches to meet this growing gap between practice and research. Momentum continues to grow in the area of narrative and arts-based methods, as evidenced by recent studies [12-15]. A systematic review guided by inclusive conceptualization of the visual arts, that includes the common artistic approach of narrative storytelling, will help inform this innovative area of investigation.

Extant literature on arts-based methods in healthcare is abundant, yet knowledge gaps exist. A literature review of a subset of the arts-based literature (up until 2009) was recently published in *Arts* &*Health*[22]. The parameters of this review did not include the use of narrative storytelling approaches in healthcare [12] and focused largely on arts-based approaches for data generation. The findings from this review illustrated further need for clarification regarding the utility of arts-based methods. Narrative storytelling as a research modality has received increasing recognition in healthcare [12-15]; however, the use of narrative storytelling and other arts-based approaches to facilitate knowledge translation is considered novel. Understanding the ways in which narrative storytelling, as well as visual art, are used in healthcare [23-25] is necessary in order to identify venues for their xutility in the clinical setting [26-29] and to develop approaches for their use as vehicles to translate research into healthcare consumers’ decision-making [30], healthcare professionals’ practice [31], and decision-makers’ policy. This project will address these gaps through a systematic review intended to identify the current uses and effectiveness of these approaches in the healthcare arena.

Narrative storytelling is one of the oldest forms of communication and is recognized in many professional disciplines as being an effective means of conveying information, understanding personal experiences, and increasing memory retention [6]. Various meanings surround the term ‘narrative’ in healthcare research. This has occurred in part because narrative is used within healthcare in different ways, such as for diagnosis, therapy, as a research method, and as a knowledge translation tool to communicate complex health information. Most commonly, narrative inquiry is regarded as an effective means for understanding the complex individual experience of health and illness [31]. The frequent use of narrative as a research method, in both data collection and analysis [23,24,31] is testimony to one aspect of its utility. However, there is untapped potential for alternative uses of narrative, such as a knowledge translation strategy to communicate complex health related information to a variety of stakeholders (patients, families, healthcare providers, decision makers). For the purpose of this study, narrative storytelling will be conceptualized as any means of providing information in a story format and the term is used interchangeably with storytelling.

Theoretically speaking, arts-based research methods can be utilized at each stage of the research process. As a result, terminology regarding arts-based methods can be varied, with the terms ‘arts-based approaches’ [32,33], ‘arts-based methods’ [34,35], and ‘arts-based research methods’ [36] commonly being used interchangeably. Further, although similarity between the types of arts-based methods exists, the indications for their use in research greatly differ. In order to generate a clear conceptual definition, for the purposes of this study ‘arts-based approaches’ will be operationalized as the use of visual representation (for example, painting, drawing, photography, sculpture).

The purpose of this study is to systematically identify and synthesize current research on narrative storytelling and visual art to translate and disseminate research in healthcare. The findings from this research project will provide critical information for: (1) researchers conducting KT intervention studies; (2) nursing, medicine, and allied healthcare professionals; (3) healthcare consumers, including patients and families; and (4) decision makers and knowledge users who are charged to increase use of the latest research in healthcare settings.

## Methods

### Objectives and key questions

The objectives for this systematic review are to: (1) systematically locate, assess and report on studies that have used narrative storytelling and/or visual art to disseminate research findings with a variety of audiences/users; (2) describe the narrative storytelling and/or visual art approaches used including attributes of the narrative/arts-based methods, effectiveness and the modifying factors/variables relevant to the respective context; (3) evaluate the narrative storytelling and visual art used in terms of changes at the healthcare system, health provider and/or patient/consumer/family level; and (4) identify possible strategies to facilitate future use of narrative storytelling and visual art as approaches to translate research to multiple audiences.

In accordance with the project’s aim and objectives, the following questions will guide this project: (1) what is the ‘state of the science’ for the use of narrative storytelling and visual art to communicate, disseminate and/or transfer research information to multiple audiences (for example, healthcare consumers, healthcare professionals, policy makers)? And (2) what is the effectiveness of narrative storytelling and visual art to translate and/or disseminate research in healthcare?

### Methodology

The systematic review will follow a comprehensive process using rigorous methodological guidelines to synthesize diverse forms of research evidence [37]. Our methods will largely be informed by the conventional approach to systematic reviews and will be supplemented to accommodate the nature of the literature (for example, narrative storytelling and visual art approaches) and different study designs (for example, randomized controlled trials (RCTs), qualitative studies). Although a degree of controversy exists surrounding the legitimacy of synthesizing various research methodologies (for example, quantitative and qualitative), an exclusive reliance on research studies employing RCTs, controlled clinical trials (CCTs), controlled before and after (CBA) studies, and interrupted time series (ITS) designs may not reflect the intricacies of the different types of ‘evidence’ utilized to guided decision-making [38]. There is growing recognition, particularly in the policy sector, that the complexities of evidence cannot be captured exclusively through one methodology [37,39,40]. Therefore, to respond to the needs of ‘decision makers’ and to acknowledge the diverse landscape in which the narrative storytelling and visual-arts-based literature resides, the methodological assumption guiding this project is one of inclusivity rather than exclusivity.

### Literature search

In collaboration with the research team, a health research librarian (with information science training) will design and implement search strategies (Additional file 1) to identify evidence. Previous systematic review work completed by members of this group (for example, SDS and LH) will inform our search for studies to be included in the current review. With this foundation, we will work with the health research librarian to refine and test our search strategy parameters. To ensure an exhaustive search is conducted, a comprehensive set of subject headings and keywords that will be used in a variety of databases will be generated. The search strategy will be guided by language (English) and date (1990 to 2011) restrictions. The decision to restrict to English studies was informed by recent systematic research evidence that indicated no empirical evidence of bias if papers written in languages other than English (LOE) are excluded [41]. The date restrictions reflect the emergence of research on arts-based approaches in healthcare [42] and were purposively selected to capture all relevant literature. The following electronic databases will be systematically searched: PubMed, Scopus, Ovid MEDLINE, Cochrane Central Register of Controlled Trials, Cochrane Database of Systematic Reviews, Database of Abstracts of Reviews of Effects, Health Technology Assessment Database, HealthStar, EMBASE (Excerpta Medica), CINAHL (Cumulative Index to Nursing and Allied Health Literature), PsycINFO (Psychological Abstracts), ERIC and Sociological Abstracts. We will also identify relevant dissertations, and search the reference lists of included studies for relevant citations.

### Study inclusion criteria

Studies will not be excluded based upon research design. The inclusion of a range of study designs is particularly important in an emerging field such as the use of narrative storytelling and visual-arts-based approaches. The merit in including these designs is that the results of our systematic review will reflect the rich and emerging literature base in this field as well as generate hypotheses that could be tested in studies with more rigorous designs. The inclusion criteria shown in Table 1 will be used for study selection.

**Table 1:** Inclusion criteria

**Study design**	**Primary research studies of all designs (for example, experimental, quasi experimental, and non**-**experimental designs)**
Context	The study focuses one of the following:
	(1) treatment and management of illness;
	(2) preservation of mental and physical wellbeing; or
	(3) services offered by medical and/or allied health professionals and trainees
Interventions	Narrative storytelling and/or visual art used with the primary purpose of disseminating/translating research or enhancing research uptake
Outcomes	Empirically evaluated or assessed change at the professional/process, patient or economic level

### Study selection

A two-step process will be used for study screening. Titles and abstracts (when available) will be independently screened against the inclusion criteria by two trained reviewers. Each article will be classified as ‘include’, ‘exclude’, or ‘unclear’. The full text of articles classified as ‘include’ or ‘unclear’ will be retrieved, and each will be independently reviewed against predetermined inclusion criteria (Additional file 2), using a standard form. A third-party adjudicator will be utilized for discrepancies unresolved by dialogue between the two reviewers.

### Quality assessment

The criteria for assessing the methodological quality of included studies will be guided by study design. Included studies will be independently assessed by two independent reviewers. Discrepancies will be addressed through discussion or third-party adjudication. Inter-rater agreement will be calculated using the weighted κ statistic [43]. The methodological quality of included quantitative studies will be assessed using the Quality Assessment Tool for Quantitative Studies (Additional file 3) [44]. This tool has been evaluated for content and construct validity and inter-rater reliability and meets accepted standards [45]. The results from the tool will lead to an overall methodological rating of strong, moderate or weak in eight sections: selection bias, study design, confounders, blinding, data collection methods, withdrawals/dropouts, intervention integrity; and analysis. The methodological quality of qualitative studies will be assessed using the Quality Assessment Tool for Qualitative Studies (Additional file 4) [46]. This tool assesses the aims of the research, research methods and design, collection and analysis, results, discussion and conclusions. This tool was selected because of structural similarities to the quantitative tool that will facilitate ease of presenting the individual quality criteria and facilitate study comparison. Given the emerging state of the field, studies will not be excluded based on the quality assessment rating.

### Data extraction

Study data will be extracted using standard forms (Additional file 5) and entered into MS Excel spreadsheets in tabular form (Microsoft; Redmond, WA, USA). Data will be extracted by one reviewer and checked for accuracy and completeness by a second reviewer. Data to be extracted include: study design and process, participant characteristics, description of the narrative storytelling and visual-arts-based approaches, ethical considerations and study findings. The data extraction form will be trialed on ten studies to refine the form and ensure the form captures all of the intricacies of both qualitative and quantitative designs.

### Data analysis/synthesis

Foremost, the data extracted from research articles that describe the use of visual art and narrative storytelling approaches to KT will be grouped and analyzed by study design (for example, qualitative, RCT, CBA, and so on), and type of approach (narrative storytelling and/or visual art). From this analysis we will present a descriptive analysis of the included studies and look at the patterns in terms of the effectiveness of the narrative storytelling and visual arts as KT strategies.

Evidence tables will be created that describe the studies included in the review. Variables to be described in the analysis include: (1) country of primary author, (2) study design, (3) quality of studies, (4) type and details of narrative storytelling and visual art, (5) patient population, (6) setting, (7) purpose/objectives of the intervention/study, (8) outcomes assessed, and (9) results. A qualitative review of the studies across the narrative storytelling and visual-arts-based approaches will allow us to not only examine what approaches are successful, but evaluate what it is about different strategies that may work, for whom, and under what circumstances [47]. The value of our review is that data will not just be pooled to get an overall assessment of whether visual art and narrative storytelling approaches are effective, but rather assess the intricacies and details of these approaches. If there is sufficient clinical and statistical homogeneity across groups of studies employing randomized control designs and assessing similar outcomes, we will perform meta-analyses in Review Manager (RevMan 5.1) [48] using standard methods [49].

### Integrated knowledge translation plan

#### Decision-maker and stakeholder partnerships

Our strategically created multidisciplinary research team of KT researchers, systematic review experts, clinicians, and decision makers is based on the Linkage and Exchange Model [50]. We purposefully decided to have a small investigative team with a broad range of complementary expertise to facilitate efficient teamwork and achievement of project timelines. All research team members will participate in regularly scheduled teleconferences and/or in-person meetings held every 3 months focusing on project progress and discussion of project findings. As a group, we have a history of strong collaborative relationships that will translate into efficiency and productivity.

Knowledge users by virtue of their roles and professional responsibilities are challenging to involve in the day-to-day operations of a knowledge synthesis grant. As a team, we acknowledged this and we developed a strong Knowledge User Advisory Panel to ensure that our synthesis outputs respond to the information needs of knowledge users and stakeholders. Eight knowledge users reflect the multiple and relevant end-users and audiences for this project and we will utilize the expertise of the Knowledge User Advisory Panel as needed, to provide strategic advice. The research team and Knowledge User Advisory Panel will formally meet via teleconference on two occasions, at the midpoint of the project and the end of the project. The Advisory Panel is comprised of healthcare consumers, artists, writers, healthcare professionals, and decision makers with a vested interest in novel approaches to engage and communicate with multiple audiences. The Knowledge User Advisory Panel will advise the research team on the strategic development of suitable ‘end products’ of the systematic review for planned dissemination to the appropriate local, national and international groups and associations. Meaningful engagement with users of research by means of our Knowledge User Advisory Panel and our ongoing consultations with stakeholders from a broad range of arts organizations will ensure that: (1) our research questions and project aims are relevant from the outset and applicable to issues of concerns to them locally, (2) project funds are used judiciously, and (3) the findings inform innovative strategies to make a quality difference in clinical practice, education and research endeavors in terms of using original and effective narrative storytelling and visual-arts-based approaches in health.

In addition to having an engaged Knowledge User Advisory Panel to facilitate strategic networking and dissemination of findings from the study, members of our research team are members of important and strategic networks, which will provide useful platforms for engaging with multiple audiences. Our collaborative interactions with provincial and national associations from several sectors (arts, health), and the engagement and hands-on involvement of a Knowledge User Advisory Panel representing multiple audiences ensures that the findings are policy relevant and that recommendations are appropriate and achievable in the clinical, educational and policy sectors.

#### Outcomes: end-of-grant knowledge translation

Our knowledge dissemination plan is based on the Canadian Health Services Research Foundation Model [51] and the research of Lavis and colleagues [52]. We will customize the research results to targeted user groups: healthcare consumers, artists/writers, healthcare professionals, decision makers and researchers. We will disseminate the findings from our systematic review in ways that are congruent with our SR findings. We will work closely with our Advisory Panel to actively engage and share our findings with key healthcare consumer groups and artist and writer venues.

We will present at healthcare research seminars and conferences, provide specific fact sheets, and meet face-to-face or communicate by phone to discuss the findings from the project. We will highlight practical strategies that could maximize use of non-traditional approaches in their specific setting. We will also circulate a one-page executive summary and project technical report that addresses the project’s objectives.

## Abbreviations

CBA: Controlled before and after design; CCT: Clinical controlled trial; ITS: Interrupted time series design; KT: Knowledge translation; RCT: Randomized controlled trial.

## Competing interests

The authors declare that they have no competing interests.

## Author’s information

SDS holds a CIHR New Investigator Award and Population Health Investigator Award from the Alberta Heritage Foundation for Medical Research (now Alberta Innovates - Health Solutions). MA is a PhD student in the Faculty of Nursing and currently holds a Canadian Child Health Clinician-Scientist (CCHCSP) Studentship, an Alberta Registered Nurses Educational Trust (ARNET) President’s Scholarship (Doctoral level), and a Women & Children’s Health Research Institute (WCHRI) Studentship. LH holds a CIHR New Investigator Award.

## Authors’ contributions

SDS conceptualized this study and secured study funding from the Canadian Institutes for Health Research (CIHR). She led and designed this study. PBM, LH and MA assisted with the study design. All authors contributed to the manuscript drafts and reviewed the final manuscript.

## Supplementary Material

Additional file 1 Literature search: use of narrative and arts-based approaches in healthcare. Click here for file

Additional file 2 Second level screening form. Click here for file

Additional file 3 Quality Assessment Tool for Quantitative Studies. Click here for file

Additional file 4 Quality Assessment Tool for Qualitative Studies. Click here for file

Additional file 5 Data extraction form. Click here for file
