# Supplementary material for: Protocol for a systematic review of the use of narrative storytelling and visual-arts-based approaches as knowledge translation tools in healthcare
Source: Syst Rev. 2013 Mar 20;2:19. doi: 10.1186/2046-4053-2-19 (PMC3627614; doi:10.1186/2046-4053-2-19)
Supplement: Additional file 1 — Literature search: use of narrative and arts-based approaches in healthcare. [file 2046-4053-2-19-S1.docx]

**LITERATURE SEARCH—Use of Narrative and Arts-Based Approaches in Healthcare**

Search Summary:

| **Review** | **Date Searched** | **Number Retrieved** | **After Duplicate Removal** |
| --- | --- | --- | --- |
| Medline | 22 May 2012 | 3,000 | 3,000 |
| Cochrane Library:  CCTR:  CDSR, DARE, HTA | 22 May 2012  22 May 2012 | 14  185 | 8  181 |
| ERIC | 22 May 2012 | 1,570 | 1,529 |
| PsycINFO | 23 May 2012 | 1,722 | 1,441 |
| HealthStar | 23 May 2012 | 2,680 | 15 |
| PubMed | 23 May 2012 | 8 | 3 |
| Cinahl | 23 May 2012 | 1,839 | 1,759 |
| Sociological Abstracts | 25 May 2012 | 588 | 522 |
| Web of Science | 25 May 2012 | 1,385 | 897 |
| Biosis Previews | 25 May 2012 | 1,446 | 1,191 |
| **Total** |  | **14,437** | **10,546** |

Database: Medline via Ovid <1946 to Present>

Search Title: Storytelling for KT --narrowed terms-- _ sans radio_tv_movies--1.2| Medline -- 26 April 2012 -- AM

Date Searched: 14 May 2012 (original search); 22 May 2012 (final search)

Results: 3,025; 3,000 after Ovid duplicate removal function

| *MeSH and text terms to capture arts and narrative:*  1. art/  2. cartoons as topic/  3. paintings/  4. sculpture/  5. Literature/  6. Drama/  7. Medicine in Literature/  8. Poetry as Topic/  9. Narration/  10. Books/  11. Books, Illustrated/  12. Writing/  13. Photography/  14. Music/  15. Dancing/  16. ((art or arts or artist*) adj5 (health* or medicine)).tw.  17. ((paint* or draw* or drew) adj5 (health* or medicine)).tw.  18. ((picture* or illustration* or sculpture* or cartoon* or comic* or photo* or graphic*) adj5 (health* or medicine)).tw.  19. (camera* adj5 (health* or medicine)).tw.  20. ((narrative* or narration* or story* or stories or book* or writing* or poem* or poet*) adj5 (health* or medicine)).tw.  21. ((drama* or theatre or theater or theatric* or actor* or actress*) adj5 (health* or medicine)).tw.  22. ((dance or dancing or music*) adj5 (health* or medicine)).tw. |
| --- |
| **23. or/1-22** [combination of MeSH and index terms r/t art/narrative] (74,120) |
| *MeSH and text word terms to capture knowledge translation:*  24. "Diffusion of Innovation"/  25. exp Health Promotion/  26. Health Communication/  27. Health Education/  28. Information Dissemination/  29. Public Health/  30. (educat* adj1 (program* or clinic*)).tw.  31. ((physician* or doctor* or nurse* or pharm* or "health care profession*" or research* or patient* or client* or consumer*) adj1 (educat* or teach* or instruct* or inform* or program* or intervention* or outcome*)).tw.  32. ((research or knowledge or evidence) adj2 ("use" or utiliz* or adopt* or implement* or disseminat* or uptake or transfer* or translat* or support*)).tw.  33. (innovat* adj3 adopt*).tw. |
| **34. or/24-33** [combination of MeSH and index terms r/t knowledge translation] (321,080) |
| **35. and/23,34** [combination of art/narrative + knowledge translation results] (3,231) |
| *Publication types to omit from the search results:*  36. (comment or editorial or letter or news or newspaper article).pt. [publication types to be excluded from search] (1,276,735) |
| **37. 35 not 36** [removal of unwanted publication types] (3,052)  38. remove duplicates from 37 (3,000) |

Database Searched: Evidence Based Medicine Reviews via Ovid **Cochrane Central Register of Controlled Trials** <April 2012>

Search Title: Storytelling for KT 2.0A | Cochrane -- CCRT -- 9 May 2012 -- AM

Date Searched: 9 May 2011 (original search); 22 May 2012 (final search)

Results: 14

| *MeSH and text terms to capture arts and narrative:*  1. art/  2. cartoons as topic/  3. paintings/  4. literature/  5. drama/  6. medicine in literature/  7. poetry as topic/  8. narration/  9. books/  10. books, illustrated/  11. writing/  12. photography/  13. music/  14. Dancing/  15. ((art or arts or artist*) adj5 (health* or medicine)).tw.  16. ((paint* or draw* or drew) adj5 (health* or medicine)).tw.  17. ((picture* or illustration* or sculpture* or cartoon* or comic* or photo* or graphic*) adj5 (health* or medicine)).tw.  18. (camera* adj5 (health* or medicine)).tw.  19. ((narrative* or narration* or story* or stories or book* or writing* or poem* or poet*) adj5 (health* or medicine)).tw.  20. ((drama* or theatre or theater or theatric* or actor* or actress*) adj5 (health* or medicine)).tw.  21. ((dance or dancing or music*) adj5 (health* or medicine)).tw. |
| --- |
| 22. **or/1-21** [combination of MeSH and index terms r/t art/narrative] (1,572) |
| *MeSH and text word terms to capture knowledge translation:*  23. exp "Diffusion of Innovation"/  24. exp health promotion/  25. health communication/  26. exp health education/  27. information dissemination/  28. public health/  29. (educat* adj1 (program* or clinic*)).tw.  30. ((physician* or doctor* or nurse* or pharm* or "health care profession*" or research* or patient* or client* or consumer*) adj1 (educat* or teach* or instruct* or inform* or program* or intervention* or outcome*)).tw.  31. ((research or knowledge or evidence) adj2 ("use" or utilize or adopt* or implement* or disseminat* or uptake or transfer* or translat* or support*)).tw.  32. (innovat* adj3 adopt*).tw. |
| 33. **or/23-32** [combination of MeSH and index terms r/t knowledge translation] (27,036) |
| 34. **and/22,33** [combination of art/narrative + knowledge translation results] (215) |
| 35. limit 34 to medline records (201)  36. **34 not 35** [removal of Medline records for search results] (14) |

Database Searched: Evidence Based Medicine Reviews via Ovid

**Cochrane Database of Systematic Reviews** <2005 to November 2011>, **Database of Abstracts of Reviews of Effects** <4th Quarter 2011>, **Health Technology Assessment** <4th Quarter 2011>

Search Title: Storytelling for KT 2.0B | Cochrane -- CDSR DARE HTA -- 9 May 2012 -- AM

Date Searched: 9 May 2012 (original search); 22 May 2012 (final search)

Results: 185

| *Text terms to capture arts and narrative:*  1. ((art or arts or artist*) adj5 (health* or medicine)).mp.  2. ((paint* or draw* or drew) adj5 (health* or medicine)).mp.  3. ((picture* or illustration* or sculpture* or cartoon* or comic* or photo* or graphic*) adj5 (health* or medicine)).mp.  4. (camera* adj5 (health* or medicine)).mp.  5. ((narrative* or narration* or story* or stories or book* or writing* or poem* or poet*) adj5 (health* or medicine)).mp.  6. ((drama* or theatre or theater or theatric* or actor* or actress*) adj5 (health* or medicine)).mp.  7. ((dance or dancing or music*) adj5 (health* or medicine)).mp. |
| --- |
| 8. **or/1-7** [arts and narrative] (273) |
| *Text word terms to capture knowledge translation:*  9. ((diffusion or adopt*) adj3 innovat*).mp.  10. (information adj3 disseminat*).mp.  11. (educat* adj1 (program* or clinic*)).mp.  12. ((physician* or doctor* or nurse* or pharm* or "health care profession*" or research* or patient* or client* or consumer*) adj1 (educat* or teach* or instruct* or inform* or program* or intervention* or outcome*)).mp.  13. ((research or knowledge or evidence) adj2 ("use" or utilize or adopt* or implement* or disseminat* or uptake or transfer* or translat* or support*)).mp. |
| 14. **or/9-13** [combination of text terms to represent KT] (8,939) |
| 15. **and/8,14** [combination of terms for arts and narrative + KT] (185) |
| 16. remove duplicates from 15 (185) |

Database: ERIC via Ovid <1965 to April 2012>

Search Title: Storytelling for KT 3.2 -- further narrowed terms | ERIC -- 11 May 2012 -- AM

Search Date: 11 May 2012 (original search); 22 May 2012 (final search)

Results: 1,621; 1570 after Ovid duplicate removal function

| *Index and text terms to capture arts and narrative:*  1. art/ or art products/  2. Art Activities/  3. exp Music Activities/  4. fine arts/  5. dance/  6. exp music/  7. theater arts/ or acting/ or drama/ or dramatics/  8. visual arts/ or childrens art/ or design crafts/ or freehand drawing/ or graphic arts/ or handicrafts/ or "painting (visual arts)"/ or photography/ or portraiture/ or sculpture/  9. Literature/  10. exp artists/  11. exp poetry/  12. exp prose/  13. childrens writing/ or creative writing/ or descriptive writing/ or expository writing/ or journal writing/ or playwriting/  14. cartoons/  15. illustrations/  16. books/ or picture books/  17. personal narratives/ or narration/  18. story telling/ or oral tradition/ or reading aloud to others/ or story reading/ or tales/  19. ((art or arts or artist*) adj5 (health* or medicine)).tw.  20. ((paint* or draw* or drew) adj5 (health* or medicine)).tw.  21. ((picture* or illustration* or sculpture* or cartoon* or comic* or photo* or graphic*) adj5 (health* or medicine)).tw.  22. (camera* adj5 (health* or medicine)).tw.  23. ((narrative* or narration* or story* or stories or book* or writing* or poem* or poet*) adj5 (health* or medicine)).tw.  24. ((drama* or theatre or theater or theatric* or actor* or actress*) adj5 (health* or medicine)).tw.  25. ((dance or dancing or music*) adj5 (health* or medicine)).tw. |
| --- |
| 26. **or/1-25** [combination of index and text terms r/t arts and narration] (90,217) |
| *Index and text word terms to capture knowledge translation:*  27. information dissemination/  28. health promotion/  29. health education/  30. patient education/  31. (educat* adj3 clinic*).tw.  32. ((physician* or doctor* or nurse* or pharm* or "health care profession*" or patient* or client* or consumer*) adj1 (educat* or teach* or instruct* or inform* or program* or intervention* or outcome*)).tw.  33. ((research or knowledge or evidence) adj1 ("use" or utiliz* or adopt* or implement* or disseminat* or uptake or transfer* or translat* or support*)).tw.  34. (innovat* adj3 adopt*).tw. |
| 35. **or/27-34** [combination of index and text terms r/t KT] (51,412) |
| 36. **and/26,35** [combination of arts and narrative + KT terms] (1,796) |
| 37. (book product reviews or computer programs or opinion papers).pt. (183,476)  38. **36 not 37** [removal of unwanted publication types] (1,621)  39. remove duplicates from 38 (1,570) |

Database: PsychINFO via Ovid <1806 to May Week 2 2012>

Search Title: "Storytelling for KT 4.1 -- narrowed search terms" | PsycINFO – 11 May 2012 -- AM

Search Date: 11 May 2012 (original search); 22 May 2012 (final search)

Results: 1,731; 1,722 after Ovid duplicate removal function

| *Index and text terms to capture arts and narrative:*  1. exp art/  2. arts/  3. exp artists/  4. "Cartoons (Humor)"/  5. dance/  6. exp music/  7. singing/  8. exp Theatre/  9. exp literature/  10. Creative Writing/  11. narratives/  12. books/  13. ((art or arts or artist*) adj5 (health* or medicine)).mp.  14. ((paint* or draw* or drew) adj5 (health* or medicine)).mp.  15. ((picture* or illustration* or sculpture* or cartoon* or comic* or photo* or graphic*) adj5 (health* or medicine)).mp.  16. (camera* adj5 (health* or medicine)).mp.  17. ((narrative* or narration* or story* or stories or book* or writing* or poem* or poet*) adj5 (health* or medicine)).mp.  18. ((drama* or theatre or theater or theatric* or actor* or actress*) adj5 (health* or medicine)).mp.  19. ((dance or dancing or music*) adj5 (health* or medicine)).mp. |
| --- |
| 20. **or/1-19** [combination of index and text terms r/t arts and narrative] (63,599) |
| *Index and text word terms to capture knowledge translation:*  21. knowledge transfer/  22. information dissemination/  23. knowledge management/  24. health promotion/  25. exp health education/  26. (educat* adj1 (program* or clinic*)).mp.  27. ((physician* or doctor* or nurse* or pharm* or "health care profession*" or patient* or client* or consumer*) adj1 (educat* or teach* or instruct* or inform* or program* or intervention* or outcome*)).mp.  28. ((research or knowledge or evidence) adj1 ("use" or utiliz* or adopt* or implement* or disseminat* or uptake or transfer* or translat* or support*)).mp.  29. (innovat* adj3 adopt*).mp. |
| 30. **or/21-29** [combination of index and text words r/t KT] (96,575) |
| 31. **and/20,30** [combination of arts and narration + KT] (1,731) |
| 32. remove duplicates from 31 (1,722) |

Database: HealthSTAR via Ovid

Search Title: Storytelling for KT 5.0 | HealthStar – 11 May 2012 -- AM

Search Date: 14 May 2012

Results:

| *MeSH and text terms to capture arts and narrative:*  1. Art/  2. Cartoons/  3. Paintings/  4. Sculpture/  5. Literature/  6. Drama/  7. Medicine in Literature/  8. Poetry/  9. Narration/  10. Books/  11. Books, Illustrated/  12. Writing/  13. Photography/  14. Music/  15. Dancing/  16. ((art or arts or artist*) adj5 (health* or medicine)).tw.  17. ((paint* or draw* or drew) adj5 (health* or medicine)).tw.  18. ((picture* or illustration* or sculpture* or cartoon* or comic* or photo* or graphic*) adj5 (health* or medicine)).tw.  19. (camera* adj5 (health* or medicine)).tw.  20. ((narrative* or narration* or story* or stories or book* or writing* or poem* or poet*) adj5 (health* or medicine)).tw.  21. ((drama* or theatre or theater or theatric* or actor* or actress*) adj5 (health* or medicine)).tw.  22. ((dance or dancing or music*) adj5 (health* or medicine)).tw. |
| --- |
| 23. **or/1-22** [ccombination of MeSH and index terms r/t arts and narrative] (53,407) |
| *MeSH and text word terms to capture knowledge translation:*  24. "diffusion of innovation"/  25. Information Dissemination/  26. exp Health Promotion/  27. Health Communication/  28. Health Education/  29. Public Health/  30. (educat* adj1 (program* or clinic*)).tw.  31. ((physician* or doctor* or nurse* or pharm* or "health care profession*" or research* or patient* or client* or consumer*) adj1 (educat* or teach* or instruct* or inform* or program* or intervention* or outcome*)).tw.  32. ((research or knowledge or evidence) adj2 ("use" or utiliz* or adopt* or implement* or disseminat* or uptake or transfer* or translat* or support*)).tw.  33. (innovat* adj3 adopt*).tw. |
| 34. **or/24-33** [combination of MeSH and index terms r/t knowledge translation] (301,208) |
| 35. **and/23,34** [combination of art/narrative + knowledge translation results] (3,149) |
| 36. (comment or editorial or letter or news or newspaper article).pt. [publication types to be excluded from search] (867,274) |
| 37. **35 not 36** (2,971)  38. remove duplicates from 37 (2,680) |

Database: PubMed via NLM <last 180 days only>

Search Title: Storytelling for KT -- 15 May 2012 -- AM

Search Date: 15 May 2012 (original search); 23 May 2012 (final search)

Results: 8

| ((((((((((((("Art"[Mesh:noexp]) OR "Cartoons as Topic"[Mesh]) OR "Paintings"[Mesh]) OR "Sculpture"[Mesh])) OR (((("Literature"[Mesh:noexp]) OR "Drama"[Mesh]) OR "Medicine in Literature"[Mesh]) OR "Poetry as Topic"[Mesh])) OR ("Narration"[Mesh])) OR (("Books"[Mesh:noexp]) OR "Books, Illustrated"[Mesh])) OR ("Writing"[Mesh:noexp])) OR ("Photography"[Mesh:noexp])) OR ("Music"[Mesh])) OR ("Dancing"[Mesh]))) AND ((((((("Diffusion of Innovation"[Mesh:noexp])) OR ("Information Dissemination"[Mesh])) OR ("Health Promotion"[Mesh])) OR ("Health Communication"[Mesh])) OR ("Health Education"[Mesh:noexp])) OR ("Public Health"[Mesh:noexp])) |
| --- |

Database: CINAHL® Plus with Full Text via Ebsco <1937 to present>

#### Search Title: Storytelling for KT -- Final AB and TI only -- 15 May 2012 -- AM

Date Searched: 15 May 2012 (original search); 23 May 2012 (final search)

Results: 1,839

| S33=**S31 NOT S32** (1,839)  S32=S31 Limiters - Publication Type: Book Review, Commentary, Computer Program, Editorial, Letter (141) |
| --- |
| S31=S30 Limiters - Exclude MEDLINE records (1,980) |
| S30=**S19 and S29** (4,448) |
| S29=**S20 or S21 or S22 or S23 or S24 or S25 or S26 or S27 or S28** (223,165) |
| *Index and text word terms to capture knowledge translation:*  S28=TI (innovat* N3 adopt*) OR AB (innovat* N3 adopt*)  S27=TI (((research or knowledge or evidence) N1 ("use" or utiliz* or adopt* or implement* or disseminat* or uptake or transfer* or translat* or support*))) OR AB (((research or knowledge or evidence) N1 ("use" or utiliz* or adopt* or implement* or disseminat* or uptake or transfer* or translat* or support*)))  S26=TI (((physician* or doctor* or nurse* or pharm* or "health care profession*" or research* or patient* or client* or consumer*) N1 (educat* or teach* or instruct* or inform* or program* or intervention* or outcome*))) OR AB (((physician* or doctor* or nurse* or pharm* or "health care profession*" or research* or patient* or client* or consumer*) N1 (educat* or teach* or instruct* or inform* or program* or intervention* or outcome*)))  S25=TI ((educat* N1 (program* or clinic*))) OR AB ((educat* N1 (program* or clinic*)))  S24=(MH "Public Health")  S23=(MH "Health Education+")  S22=(MH "Health Promotion+")  S21=(MH "Information Management")  S20=(MH "Diffusion of Innovation") |
| S19=**S1 or S2 or S3 or S4 or S5 or S6 or S7 or S8 or S9 or S10 or S11 or S12 or S13 or S14 or S15 or S16 or S17 or S18** (54,112) |
| *Index and text terms to capture arts and narrative:*  S18=TI ((dance or dancing or music*) N5 (health* or medicine)) OR AB ((dance or dancing or music*) N5 (health* or medicine))  S17=TI ((drama* or theatre or theater or theatric* or actor* or actress*) N5 (health* or medicine)) OR AB ((drama* or theatre or theater or theatric* or actor* or actress*) N5 (health* or medicine))  S16=TI ((narrative* or narration* or story* or stories or book* or writing* or poem* or poet*) N5 (health* or medicine)) OR AB ((narrative* or narration* or story* or stories or book* or writing* or poem* or poet*) N5 (health* or medicine))  S15=TI ((camera*) N5 (health* or medicine)) OR AB ((camera*) N5 (health* or medicine))  S14=TI ((picture* or illustration* or sculpture* or cartoon* or comic* or photo* or graphic*) N5 (health* or medicine)) OR AB ((picture* or illustration* or sculpture* or cartoon* or comic* or photo* or graphic*) N5 (health* or medicine))  S13=TI ((paint* or draw* or drew) N5 (health* or medicine)) OR AB ((paint* or draw* or drew) N5 (health* or medicine))  S12=TI ((art or arts or artist*) N5 (health* or medicine)) OR AB ((art or arts or artist*) N5 (health* or medicine))  S11=(MH "Performing Artists")  S10=(MH "Singing")  S9=(MH "Dancing+")  S8=(MH "Music")  S7=(MH "Photography")  S6=(MH "Writing")  S5=(MH "Narratives")  S4=(MH "Literature+")  S3=(MH "Language Arts")  S2=(MH "Performing Arts")  S1=(MH "Art+") |

Database: Sociological Abstracts via ProQuest <1952 to present>

Date Searched: 18 May 2012

Results: 606

| ((SU.EXACT("Artists") OR SU.EXACT("Biographies") OR SU.EXACT("Storytelling") OR SU.EXACT("Narratives") OR SU.EXACT("Comics (Publications)") OR SU.EXACT("Folklore") OR SU.EXACT("Fine Arts") OR SU.EXACT("Visual Arts") OR SU.EXACT("Photography/Photographic") OR SU.EXACT("Myths") OR SU.EXACT("Musicians") OR SU.EXACT("Books") OR SU.EXACT("Literature") OR SU.EXACT("Music") OR SU.EXACT("Fiction") OR SU.EXACT("Art Works") OR SU.EXACT("Actors") OR SU.EXACT("Poetry") OR SU.EXACT("Dance") OR SU.EXACT("Theater Arts")) OR ab((art OR arts OR artist* OR paint* OR draw* OR drew OR picture* OR illustration* OR sculpture* OR cartoon* OR comic* OR photo* OR graphic* OR camera* OR narrative* OR narration* OR story* OR stories OR book* OR writing* OR poem* OR poet* OR drama* OR theatre OR theater OR theatric* OR actor* OR actress* OR dance OR dancing OR music*) NEAR/3 (medicine or health*))) AND (ab((physician* or doctor* or nurse* or pharm* or "health care profession*" or research* or patient* or client* or consumer*) NEAR/1 (educat* or teach* or instruct* or inform* or program* or intervention* or outcome*) OR ((research or knowledge or evidence) NEAR/2 ("use" or utiliz* or adopt* or implement* or disseminat* or uptake or transfer* or translat* or support*)) OR (innovat* NEAR/3 adopt*) OR (educat* NEAR/1 (program* or clinic*))) OR SU.EXACT("Research Applications") OR SU.EXACT("Health Education") OR SU.EXACT("Knowledge Utilization") OR SU.EXACT("Information Dissemination") OR SU.EXACT("Public Health") OR SU.EXACT("Adoption of Innovations")) |
| --- |

Database: Biosis Previews via Web of Knowledge

Date Searched: 22 May 2012 (original search); 25 May 2012 (final search)

Results: 1,447

| **# 15=#14 AND #8** (1,447) **Did not limit by document type; only removes 1 record* |
| --- |
| **# 14=#13 OR #12 OR #11 OR #10 OR #9** (241,487) |
| *Text word terms to capture knowledge translation:*  # 13=TS=((research or knowledge or evidence) NEAR/2 ("use" or utilize or adopt* or implement* or disseminat* or uptake or transfer* or translat* or support*))  # 12=TS=((physician* or doctor* or nurse* or pharm* or "health care profession*" or research* or patient* or client* or consumer*) NEAR/1 (educat* or teach* or instruct* or inform* or program* or intervention* or outcome*)) (102,836)  # 11=TS=((educat* program*) or (educat* clinic*))  # 10=TS=(information disseminat*) (3,751)  # 9=TS=((diffusion innovat*) or (adopt* innovat*)) |
| **# 8=#7 OR #6 OR #5 OR #4 OR #3 OR #2 OR #1** (17,875) |
| *Text terms to capture arts and narrative:*  # 7=TS=((dance or dancing or music*) NEAR/5 (health* or medicine))  # 6=TS=((drama* or theatre or theater or theatric* or actor* or actress*) NEAR/5 (health* or medicine))  # 5=TS=((narrative* or narration* or story* or stories or book* or writing* or poem* or poet*) NEAR/3 (health* or medicine))  # 4=TS=(camera* NEAR/5 (health* or medicine))  # 3=TS=((picture* or illustration* or sculpture* or cartoon* or comic* or photo* or graphic*) NEAR/5 (health* or medicine))  # 2=TS=((paint* or draw* or drew) NEAR/5 (health* or medicine))  # 1=TS=((art or arts or artist*) NEAR/5 (health* or medicine)) |

Database: Web of Science (Databases=SCI-EXPANDED, SSCI, A&HCI, CPCI-S, CPCI-SSH) via Web of Knowledge

Date Searched: 22 May 2012 (original search); 25 May 2012 (final search)

Results: 1,385

| # 16=#14 AND #6 Refined by: **[excluding] Document Type**=( ITEM ABOUT AN INDIVIDUAL OR BIBLIOGRAPHY OR BIOGRAPHICAL ITEM OR NOTE OR BOOK REVIEW OR EDITORIAL MATERIAL) (1,385) |
| --- |
| **# 15=#14 AND #6** (1,436) |
| **# 14=#13 OR #12 OR #11 OR #10 OR #9 OR #8 OR #7** (14,109) |
| *Text word terms to capture knowledge translation:*  # 13=TS=((dance or dancing or music*) NEAR/5 (health* or medicine))  # 12=TS=((drama* or theatre or theater or theatric* or actor* or actress*) NEAR/5 (health* or medicine))  # 11=TS=((narrative* or narration* or story* or stories or book* or writing* or poem* or poet*) NEAR/5 (health* or medicine))  # 10=TS=(camera* NEAR/5 (health* or medicine))  # 9=TS=((picture* or illustration* or sculpture* or cartoon* or comic* or photo* or graphic*)  # 8=TS=((paint* or draw* or drew) NEAR/5 (health* or medicine))  # 7=TS=((art or arts or artist*) NEAR/5 (health* or medicine)) |
| **# 6=#5 OR #4 OR #3 OR #2 OR #1** (467,463) |
| *Text terms to capture arts and narrative:*  # 5=TS=((research or knowledge or evidence) NEAR/2 ("use" or utilize or adopt* or implement* or disseminat* or uptake or transfer* or translat* or support*))  # 4=TS=((physician* or doctor* or nurse* or pharm* or "health care profession*" or research* or patient* or client* or consumer*) NEAR/1 (educat* or teach* or instruct* or inform* or program* or intervention* or outcome*))  # 3=TS=((educat* program*) or (educat* clinic*))  # 2=TS=(information disseminat*)  # 1=TS=((diffusion innovat*) or (adopt* innovat*)) |
