# Supplementary material for: Protocol for a systematic review of the use of narrative storytelling and visual-arts-based approaches as knowledge translation tools in healthcare
Source: Syst Rev. 2013 Mar 20;2:19. doi: 10.1186/2046-4053-2-19 (PMC3627614; doi:10.1186/2046-4053-2-19)
Supplement: Additional file 2 — Second level screening form. [file 2046-4053-2-19-S2.docx]

Systematic review of the use of narrative and arts-based approaches in health care

Yes No Unclear

1. Publication Type/Design: primary research study with methods & outcomes **⬜ ⬜ ⬜**
2. Context: study focuses one of the following: **⬜ ⬜ ⬜**
   1. Treatment and management of illness
   2. Preservation of mental and physical well-being
   3. Services offered by the medical and allied health professionals & trainees

3. Intervention(s): study employs “narrative” or “visual art” for one of the following: **⬜ ⬜ ⬜**

following:

- 1. As a research-based KT intervention
  2. To translate/disseminate research findings

1. Outcomes: study assesses/evaluates one of the following: **⬜ ⬜ ⬜**
   1. Health professional outcomes/process measures including change in knowledge, attitude & behaviour (e.g., number of drugs prescribed)
2. Patient outcomes including change in knowledge, attitude & behaviour (e.g., number of adverse drug events)
3. Economic outcomes (e.g., cost of intervention; changes in healthcare costs; changes in non-healthcare costs)

*One “No” means that this study no longer qualifies for inclusion in the SR.

**One “Unsure” means that this study should be filed as “Unsure.”

*** If all criteria are met, this article will be included in the SR.
