# Supplementary material for: Protocol for a systematic review of the use of narrative storytelling and visual-arts-based approaches as knowledge translation tools in healthcare
Source: Syst Rev. 2013 Mar 20;2:19. doi: 10.1186/2046-4053-2-19 (PMC3627614; doi:10.1186/2046-4053-2-19)
Supplement: Additional file 3 — Quality Assessment Tool for Quantitative Studies. [file 2046-4053-2-19-S3.docx]

**Appendix C: *Quality Assessment Tool for Quantitative Studies***

**COMPONENT RATINGS**

**A) SELECTION BIAS**

**(Q1) Are the individuals selected to participate in the study likely to be representative of the target population?**

1. Very likely
2. Somewhat likely
3. Not likely
4. Can’t tell

**(Q2) What percentage of selected individuals agreed to participate?**

1. 80 - 100% agreement
2. 60 – 79% agreement
3. less than 60% agreement
4. Not applicable
5. Can’t tell

| **RATE THIS SECTION** | **STRONG** | **MODERATE** | **WEAK** |
| --- | --- | --- | --- |
| See dictionary | 1 | 2 | 3 |

**B) STUDY DESIGN**

**Indicate the study design**

1. Randomized controlled trial
2. Controlled clinical trial
3. Cohort analytic (two group pre + post)
4. Case-control
5. Cohort (one group pre + post (before and after))
6. Interrupted time series
7. Other specify ____________________________
8. Can’t tell

**Was the study described as randomized? If NO, go to Component C.**

No Yes

**If Yes, was the method of randomization described? (See dictionary)**

No Yes

**If Yes, was the method appropriate? (See dictionary)**

| **RATE THIS SECTION** | **STRONG** | **MODERATE** | **WEAK** |
| --- | --- | --- | --- |
| **See dictionary** | 1 | 2 | 3 |

**C) CONFOUNDERS**

**(Q1) Were there important differences between groups prior to the intervention?**

1. Yes
2. No
3. Can’t tell

**The following are examples of confounders:**

1. Race
2. Sex
3. Marital status/family
4. Age
5. SES (income or class)
6. Education
7. Health status
8. Pre-intervention score on outcome measure

**(Q2) If yes, indicate the percentage of relevant confounders that were controlled (either in the design (e.g. stratification, matching) or analysis)?**

1. 80 – 100% (most)
2. 60 – 79% (some)
3. Less than 60% (few or none)
4. Can’t Tell

| **RATE THIS SECTION** | **STRONG** | **MODERATE** | **WEAK** |
| --- | --- | --- | --- |
| **See dictionary** | 1 | 2 | 3 |

**D) BLINDING**

**(Q1) Was (were) the outcome assessor(s) aware of the intervention or exposure status of participants?**

1. Yes
2. No
3. Can’t tell

**(Q2) Were the study participants aware of the research question?**

1. Yes
2. No
3. Can’t tell

| **RATE THIS SECTION** | **STRONG** | **MODERATE** | **WEAK** |
| --- | --- | --- | --- |
| **See dictionary** | 1 | 2 | 3 |

**E) DATA COLLECTION METHODS**

**(Q1) Were data collection tools shown to be valid?**

1. Yes
2. No
3. Can’t tell

**(Q2) Were data collection tools shown to be reliable?**

1. Yes
2. No
3. Can’t tell

| **RATE THIS SECTION** | **STRONG** | **MODERATE** | **WEAK** |
| --- | --- | --- | --- |
| **See dictionary** | 1 | 2 | 3 |

**F) WITHDRAWALS AND DROP-OUTS**

**(Q1) Were withdrawals and drop-outs reported in terms of numbers and/or reasons per group?**

1. Yes
2. No
3. Can’t tell
4. Not Applicable (i.e. one time surveys or interviews)

**(Q2) Indicate the percentage of participants completing the study. (If the percentage differs by groups, record the lowest).**

1. 80 -100%
2. 60 - 79%
3. less than 60%
4. Can’t tell
5. Not Applicable (i.e. Retrospective case-control)

| **RATE THIS SECTION** | **STRONG** | **MODERATE** | **WEAK** |  |
| --- | --- | --- | --- | --- |
| **See dictionary** | 1 | 2 | 3 | Not Applicable |

**G) INTERVENTION INTEGRITY**

**(Q1) What percentage of participants received the allocated intervention or exposure of interest?**

1. 80 -100%
2. 60 - 79%
3. less than 60%
4. Can’t tell

**(Q2) Was the consistency of the intervention measured?**

1. Yes
2. No
3. Can’t tell

**(Q3) Is it likely that subjects received an unintended intervention (contamination or co-intervention) that may influence the results?**

1. Yes
2. No
3. Can’t tell

**H) ANALYSES**

**(Q1) Indicate the unit of allocation (circle one)**

Community/organization institution/practice office/individual

**(Q2) Indicate the unit of analysis (circle one)**

Community/organization institution/practice office/individual

**(Q3) Are the statistical methods appropriate for the study design?**

1. Yes
2. No
3. Can’t tell

**(Q4) Is the analysis performed by intervention allocation status (i.e. intention to treat) rather than the actual intervention received?**

1. Yes
2. No
3. Can’t tell

**GLOBAL RATING**

**COMPONENT RATINGS**

Please transcribe the information from the gray boxes on pages 1-4 onto this page. See dictionary on how to rate this section.

| **A** | **Selection Bias** | **Strong** | **Moderate** | **Weak** |  |
| --- | --- | --- | --- | --- | --- |
|  |  | 1 | 2 | 3 |  |
| **B** | **Study Design** | **Strong** | **Moderate** | **Weak** |  |
|  |  | 1 | 2 | 3 |  |
| **C** | **Confounders** | **Strong** | **Moderate** | **Weak** |  |
|  |  | 1 | 2 | 3 |  |
| **D** | **Blinding** | **Strong** | **Moderate** | **Weak** |  |
|  |  | 1 | 2 | 3 |  |
| **E** | **Data Collection Method** | **Strong** | **Moderate** | **Weak** |  |
|  |  | 1 | 2 | 3 |  |
| **F** | **Withdrawals and Dropouts** | **Strong** | **Moderate** | **Weak** |  |
|  |  | 1 | 2 | 3 | Not Applicable |

**GLOBAL RATING FOR THIS PAPER (circle one):**

1 STRONG (no WEAK ratings)

2 MODERATE (one WEAK rating)

3 WEAK (two or more WEAK ratings)

With both reviewers discussing the ratings:

Is there a discrepancy between the two reviewers with respect to the component (A-F) ratings?

No Yes

If yes, indicate the reason for the discrepancy

1 Oversight

2 Differences in interpretation of criteria

3 Differences in interpretation of study

**Final decision of both reviewers (circle one): 1 STRONG**

**2 MODERATE**

**3 WEAK**
