# Supplementary material for: Protocol for a systematic review of the use of narrative storytelling and visual-arts-based approaches as knowledge translation tools in healthcare
Source: Syst Rev. 2013 Mar 20;2:19. doi: 10.1186/2046-4053-2-19 (PMC3627614; doi:10.1186/2046-4053-2-19)
Supplement: Additional file 4 — Quality Assessment Tool for Qualitative Studies. [file 2046-4053-2-19-S4.docx]

**Appendix D: *Quality Assessment Tool for Qualitative Studies***
