# Supplementary material for: Protocol for a systematic review of the use of narrative storytelling and visual-arts-based approaches as knowledge translation tools in healthcare
Source: Syst Rev. 2013 Mar 20;2:19. doi: 10.1186/2046-4053-2-19 (PMC3627614; doi:10.1186/2046-4053-2-19)
Supplement: Additional file 5 — Data extraction form. [file 2046-4053-2-19-S5.docx]

**Appendix E: Data Extraction Form**

| Reviewer | | | | | | | | | | | | | | Date | | |
| --- | --- | --- | --- | --- | --- | --- | --- | --- | --- | --- | --- | --- | --- | --- | --- | --- |
| **Systematic Review Of the Use of Narrative and Arts-Based Approaches in healthcare - Extraction Tool For Included Studies – Draft** | | | | | | | | | | | | | | | | |
| **Publication Information** | | | | | | | | | | | | | | | | |
| Study | | | | | | | | | | | | First Author | | | | |
| Date | | | | | | | | Journal | | | | | | | | |
| Country | | | | | | | | | | | | | | | | |
| **Demographics** | | | | | | | | | | | | | | | | |
| Sample Size | | | | | | | | Type Of Sample | | | | | | | | |
| Design Of Study | | | | | | | | | | | | | | | | |
| **Data Analysis** | | | | | | | | | | | | | | | | |
| Statistical Tests Used | | | | | | | | | | | | | | | | |
| Effect Of Intervention | | | | | | | | | | | | | | | | |
| Qualitative Data Analysis | | | | | | | | | | | | | | | | |
| Results | | | | | | | | | | | | | | | | |
| **Theoretical Framework For Study** | | | | | | | | | Done (Specify Which Theory) | | | | | | Not Done | |
| **Intervention/Implementation**: | | | | | | | | | | | | | | | | |
| **Type Of Intervention** (circle only one, append additional pages if more than one intervention) | | | | | | | | | | | | | | | | |
| **Narrative** | | | | | | **Arts-Based** | | | | | | |  | | | |
|  | | | | | |  | | | | | | |  | | | |
|  | | | | | |  | | | | | | |  | | | |
|  | | | | | |  | | | | | | |  | | | |
|  | | | | | |  | | | | | | |  | | | |
|  | | | | | |  | | | | | | |  | | | |
| What Was The Focus Of The Intervention? | | | | | | | | | | | | | | | | |
| **Format Of Intervention** | | | | | | | | | | | | | | | | |
| Interpersonal | |  | Audio/Visual | | | | Computer/Interactive | | | | | Multiple Media Used | | Not Clear | | Other |
| **Recipient Of Arts-Based/Narrative Intervention** | | | | | | | | | | | | | | | | |
| Individual(Type) | | | | | | | | Group (Type) | | | | | | Not Clear | | Other |
| **Deliverer Of Arts-Based/Narrative Intervention (Circle All Appropriate)** | | | | | | | | | | | | | | | | |
| Artist | | | | Researcher | | | | Management | | | | Educator | | Not Clear | |  |
| **Control Groups** | | | | | | | | | | | | | | | | |
| No Intervention | Standard Practice | | | | Control Group Received Intervention After Follow-Up | | | | | | Other Intervention (Type) | | | | | |
| **Outcomes** | | | | | | | | | | | | | | | | |
| Type Of Targeted Behaviour (Circle All That Apply) | | | | | | | | | | | | | | | | |
| Health Promotion | | | | Illness Prevention | | | | | | Illness Management | | Ordering | | Procedures | | Patient Education |
| Professional/Patient Communication | | | | Documentation | | | | | | Resource Use | | Patient Outcomes (Specify) | | | | |
| System Outcomes (Specify) | | | | | | | | | | Not Clear | | Other (Specify) | | | | |
| How Outcomes Were Measured: | | | | | | | | | | | | | | | | |
| Frequency Of Outcome Measurement: | | | | | | | | | | | | | | | | |
| Length Of Follow-Up Period: | | | | | | | | | | | | | | | | |
| Length Of Time Between End Of Intervention And Beginning Of Follow-Up/Outcome Measurement: | | | | | | | | | | | | | | | | |
| Additional Notes: | | | | | | | | | | | | | | | | |
